# Supplementary material for: Patient satisfaction among persons living with HIV/AIDS and receiving antiretroviral therapy in urban Uganda: A factor analysis
Source: PLoS One. 2023 Feb 2;18(2):e0280732. doi: 10.1371/journal.pone.0280732 (PMC9894454; doi:10.1371/journal.pone.0280732)
Supplement: S1 Table — (DOCX) [file pone.0280732.s001.docx]

Supplementary file

Table S1. Responses from 6-month survey measuring Patient Satisfaction with Health Services for 18-items from 475 HIV-Infected Patients on Antiretroviral Therapy at a Public Clinic in Urban Uganda.

| Characteristic | N = 475^1^ |
| --- | --- |
| Provider gives easy to understand information |  |
| Never | 119 (25%) |
| Sometimes | 25 (5.3%) |
| Usually | 154 (32%) |
| Always | 177 (37%) |
| Provider knows about patient medical history |  |
| Never | 58 (12%) |
| Sometimes | 56 (12%) |
| Usually | 191 (40%) |
| Always | 170 (36%) |
| Provider shows respect for what patient say |  |
| Never | 9 (1.9%) |
| Sometimes | 21 (4.4%) |
| Usually | 225 (47%) |
| Always | 220 (46%) |
| Provider listens carefully to patient |  |
| Never | 13 (2.7%) |
| Sometimes | 26 (5.5%) |
| Usually | 208 (44%) |
| Always | 228 (48%) |
| Provider explains things in an easy way |  |
| Never | 15 (3.2%) |
| Sometimes | 17 (3.6%) |
| Usually | 223 (47%) |
| Always | 220 (46%) |
| Provider sees patient as soon as needed |  |
| Never | 182 (38%) |
| Sometimes | 68 (14%) |
| Usually | 98 (21%) |
| Always | 127 (27%) |
| How comfortable patient feels while waiting |  |
| Never | 53 (11%) |
| Sometimes | 55 (12%) |
| Usually | 153 (32%) |
| Always | 214 (45%) |
| Provider spends enough time with patient |  |
| Never | 25 (5.3%) |
| Sometimes | 24 (5.1%) |
| Usually | 205 (43%) |
| Always | 221 (47%) |
| Provider sees patient within one hour of arrival |  |
| Never | 243 (51%) |
| Sometimes | 51 (11%) |
| Usually | 81 (17%) |
| Always | 100 (21%) |
| Patient willing to adhere to my medical regimen |  |
| Strongly disagree | 0 (0%) |
| Disagree | 1 (0.2%) |
| Agree | 222 (47%) |
| Strongly Agree | 251 (53%) |
| Unknown | 1 |
| Patient willing to return to this facility for care next time |  |
| Strongly disagree | 1 (0.2%) |
| Disagree | 1 (0.2%) |
| Agree | 232 (49%) |
