# Supplementary material for: Patient satisfaction among persons living with HIV/AIDS and receiving antiretroviral therapy in urban Uganda: A factor analysis
Source: PLoS One. 2023 Feb 2;18(2):e0280732. doi: 10.1371/journal.pone.0280732 (PMC9894454; doi:10.1371/journal.pone.0280732)
Supplement: S2 Table — Initial results with 18 items and final selection with 15 items included in the exploratory factor analysis. (DOCX) [file pone.0280732.s002.docx]

SUPPLEMENTARY FILE

Table S2. Individual and Overall Kaiser’s Measure of Sampling Adequacy. Initial results with 18 items and final selection with 15 items included in the exploratory factor analysis

|  |  |  |
| --- | --- | --- |
| **Variable** | Kaiser’s Measure of Sampling Adequacy 18 items | Kaiser’s Measure of Sampling Adequacy 15 items |
| **Overall** | 0.55 | 0.94 |
| Patient gets an answer to the medical problem the day of the visit | 0.18 | NE |
| Provider sees patient within one hour of arrival | 0.56 | 0.91 |
| Provider saw patient as soon as needed | 0.55 | 0.90 |
| How comfortable patient feels while waiting in the facility | 0.51 | 0.97 |
| Provider explain things in an easy way | 0.60 | 0.95 |
| Provider listen carefully to patient | 0.59 | 0.94 |
| Patient talk about provider about their concerns | 0.17 | NE |
| Provider gives patient easy to understand information | 0.58 | 0.94 |
| Provider knows about patient medical history | 0.57 | 0.93 |
| Provider spends enough time with patient | 0.61 | 0.96 |
| Provider shows respect for what patient say | 0.62 | 0.96 |
| Clerks are helpful | 0.58 | 0.92 |
| Clerks treat patient with courtesy and respect | 0.63 | 0.93 |
| Clerks are efficient from check-in to check-out | 0.60 | 0.96 |
| Patient willing to recommend this facility to family and friends | 0.36 | 0.98 |
| Patient willing to return to this facility for care next time | 0.58 | 0.89 |
| Patient willing to adhere to my medical regimen | 0.61 | 0.90 |
| Quality of care and services received | 0.36 | NE |

NE: Not Estimated/ excluded due to low KMO <0.40
