# Supplementary material for: Patient satisfaction among persons living with HIV/AIDS and receiving antiretroviral therapy in urban Uganda: A factor analysis
Source: PLoS One. 2023 Feb 2;18(2):e0280732. doi: 10.1371/journal.pone.0280732 (PMC9894454; doi:10.1371/journal.pone.0280732)
Supplement: S1 Questionnaire — (PDF) [file pone.0280732.s003.pdf]

Pilot for Patient Satisfaction with Health Services

CAHPS Clinician & Group Surveys : 6-Month Survey

4598

Paticipant ID ☐☐☐

Interview Date ☐☐ / ☐☐ / ☐☐☐☐ (dd / mm / yyyy)

Interview Start Time ☐☐ : ☐☐ **01=AM 02=PM**

1.Our records show that you got care from the facility named below in the last 6 months

Name of facility

☐

Is that right? ☐☐ **01=Yes 02=No**

2. Is this the facility you usually come to if you need a check-up, want advice about a health problem or when you are sick?

☐☐ **01=Yes 02=No**

3. How long have you been receiving care at this facility?

- ☐ Less than 6 months
- ☐ At least 6 months but less than 1 year
- ☐ At least 1 year but less than 3 years
- ☐ At least 3 years but less than 5 years
- ☐ 5 years or more

4. In the last 6 months, how many times did you visit this facility to get care for yourself?

- ☐ None
- ☐ 1 time
- ☐ 2
- ☐ 3
- ☐ 4
- ☐ 5 to 9
- ☐ 10 or more times

5. In the last 6 months, did you come to this facility with a medical problem during regular office hours?

☐☐ **01=Yes 02=No**

6. In the last 6 months, when you came to this facility during regular office hours, how often did you get an answer to your medical problem that very day?

- ☐ Never
- ☐ Sometimes
- ☐ Usually
- ☐ Always

7. Wait time for care includes time spent in the waiting room and exam room. In the last 6 months, how often did you see this provider **within one hour** of your arrival?

- ☐ Never
- ☐ Sometimes
- ☐ Usually
- ☐ Always

8. In the last 6 months, when you needed to see a provider, how often did you see a provider as soon as you needed ?

- ☐ Never
- ☐ Sometimes
- ☐ Usually
- ☐ Always

9. In the last 6 months, when you came to receive your medical treatment, did you feel comfortable while waiting in the facility?

- ☐ Never
- ☐ Sometimes
- ☐ Usually
- ☐ Always

10. In the last 6 months, how often did this provider explain things in a way that was easy to understand?

- ☐ Never
- ☐ Sometimes
- ☐ Usually
- ☐ Always

11. In the last 6 months, how often did this provider listen carefully to you?

- ☐ Never
- ☐ Sometimes
- ☐ Usually
- ☐ Always

12. In the last 6 months, did you talk with this provider about any health questions or concerns ?

☐☐ **01=Yes 02=No**

Int Initials: ☐☐	Comp Date:	☐☐ / ☐☐ /	2 0 ☐☐
Rev Initials: ☐☐	Rev Date:	☐☐ / ☐☐ /	2 0 ☐☐
CD Initials: ☐☐	CD Date:	☐☐ / ☐☐ /	2 0 ☐☐
		Day / Month /	Year

13. In the last 6 months, how often did this provider give you easy to understand information about these health questions or concerns?
    - ☐ Never
    - ☐ Sometimes
    - ☐ Usually
    - ☐ Always

14. In the last 6 months, how often did this provider seem to know the important information about your medical history?
    - ☐ Never
    - ☐ Sometimes
    - ☐ Usually
    - ☐ Always

15. In the last 6 months, how often did this provider show respect for what you had to say?
    - ☐ Never
    - ☐ Sometimes
    - ☐ Usually
    - ☐ Always

16. In the last 6 months, how often did this provider spend enough time with you?
    - ☐ Never
    - ☐ Sometimes
    - ☐ Usually
    - ☐ Always

17. Using any number from 0 to 10, where 0 is the worst facility possible and 10 is the best facility possible, what number would you use to rate this facility?
    - ☐ 0, Worst provider possible
    - ☐ 1
    - ☐ 2
    - ☐ 3
    - ☐ 4
    - ☐ 5
    - ☐ 6
    - ☐ 7
    - ☐ 8
    - ☐ 9
    - ☐ 10, Best provider possible

18. I am willing to recommend this facility to family and friends
    - ☐ Strongly disagree
    - ☐ Disagree
    - ☐ Agree
    - ☐ Strongly agree

19. I am willing to return to this facility for care next time
    - ☐ Strongly disagree
    - ☐ Disagree
    - ☐ Agree
    - ☐ Strongly agree

20. I am willing to adhere to my medical regimen
    - ☐ Strongly disagree
    - ☐ Disagree
    - ☐ Agree
    - ☐ Strongly agree

21. Overall, would you rate the quality of care and services received during this hospital stay as:
    - ☐ Excellent
    - ☐ Very good
    - ☐ Good
    - ☐ Fair
    - ☐ Poor

22. In the last 6 months, how often were clerks at this facility as helpful as you thought they should be?
    - ☐ Never
    - ☐ Sometimes
    - ☐ Usually
    - ☐ Always

23. In the last 6 months, how often did clerks at this facility treat you with courtesy and respect?
    - ☐ Never
    - ☐ Sometimes
    - ☐ Usually
    - ☐ Always

24. In the last 6 months, how often were the clerks at this facility efficient from check-in through check-out?
    - ☐ Never
    - ☐ Sometimes
    - ☐ Usually
    - ☐ Always

Int Initials:	[ ][ ]	Comp Date:	[ ][ ] /	[ ][ ] /	2 0 [ ][ ]
Rev Initials:	[ ][ ]	Rev Date:	[ ][ ] /	[ ][ ] /	2 0 [ ][ ]
CD Initials:	[ ][ ]	CD Date:	[ ][ ] /	[ ][ ] /	2 0 [ ][ ]
			Day	Month	Year

Pilot for Patient Satisfaction with Health Services

CAHPS Clinician & Group Surveys : 6-Month Survey

Participant ID [ ][ ][ ]

25. In general, how would you rate your overall health?
   - ☐ Excellent
   - ☐ Very good
   - ☐ Good
   - ☐ Fair
   - ☐ Poor

26. In general, how would you rate your overall **mental or emotional** health?
   - ☐ Excellent
   - ☐ Very good
   - ☐ Good
   - ☐ Fair
   - ☐ Poor

27. What is your age?

   [ ][ ] **Years old**

28. Are you male or female?

   [ ][ ] **01=Male**
   02=Female

29. What is the highest level of school that you have completed?
   - ☐ None
   - ☐ Primary 1 to 7
   - ☐ Senior 1 to 6
   - ☐ University or tertiary college

30. What is your marital status?
   - ☐ Single, never married
   - ☐ Currently married
   - ☐ Previously married (divorced/ separated/ widowed)

31. Please tell us how this facility could have improved the care and services you received in the last 6 months

   [ ]

32. How sure are you that you will be able to take all or most of the medications as directed?
   - ☐ Not at all sure
   - ☐ Somewhat sure
   - ☐ Very sure
   - ☐ Extremely sure

33. During the past 4 days, on how many days have you missed taking all your doses?
   - ☐ None
   - ☐ One day
   - ☐ Two days
   - ☐ Three days
   - ☐ Four days

34. Most anti-HIV medications need to be taken on a schedule, such as "2 times a day" or "3 times a day" or "every 8 hours". How cloely did you follow your specific schedule over the last four days?
   - ☐ Never
   - ☐ Some of the time
   - ☐ About half of the time
   - ☐ Most of the time
   - ☐ All of the time

35. When was the last time you missed any of your medications?
   - ☐ Within the past week
   - ☐ 1-2 weeks ago
   - ☐ 2-4 weeks ago
   - ☐ 1-3 months ago
   - ☐ More than 3 months ago
   - ☐ Never skip medications or not applicable

THANK YOU

To the Interviewer

Time Interview ended

[ ][ ] : [ ][ ] **01=AM**
02=PM

How long does the interview take?

[ ][ ][ ] **Minutes**

Do you think the participant was competent to answer the questions?

[ ][ ] **01=Yes**
02=No

Int Initials: [ ][ ]	Comp Date:	[ ][ ] / [ ][ ] /	[2][0][ ][ ]
Rev Initials: [ ][ ]	Rev Date:	[ ][ ] / [ ][ ] /	[2][0][ ][ ]
CD Initials: [ ][ ]	CD Date:	[ ][ ] / [ ][ ] /	[2][0][ ][ ]
		Day / Month	Year
