# Supplementary material for: Patient satisfaction among persons living with HIV/AIDS and receiving antiretroviral therapy in urban Uganda: A factor analysis
Source: PLoS One. 2023 Feb 2;18(2):e0280732. doi: 10.1371/journal.pone.0280732 (PMC9894454; doi:10.1371/journal.pone.0280732)
Supplement: Attachment — Submitted filename: Response to Reviewers-Final.docx [file pone.0280732.s004.docx]

PONE-D-21-27071

**Response to General Journal Comments**

1. Please ensure that your manuscript meets PLOS ONE's style requirements, including those for file naming. The PLOS ONE style templates- Response: This has been corrected according to journal recommendations- thank you

2. We note that the grant information you provided in the ‘Funding Information’ and ‘Financial Disclosure’ sections do not match.

When you resubmit, please ensure that you provide the correct grant numbers for the awards you received for your study in the ‘Funding Information’ section. Response: The internal seed grants at the university of Georgia are not assigned specific grant numbers**.**

3. Thank you for stating the following financial disclosure: 'JNS and KO received an internal seed grant from the University of Georgia.
The funders had no role in study design, data collection and analysis, decision to publish, or preparation of the manuscript."

We note that one or more of the authors is affiliated with the funding organization, indicating the funder may have had some role in the design, data collection, analysis or preparation of your manuscript for publication; in other words, the funder played an indirect role through the participation of the co-authors. If the funding organization did not play a role in the study design, data collection and analysis, decision to publish, or preparation of the manuscript and only provided financial support in the form of authors' salaries and/or research materials, please do the following:

a. Review your statements relating to the author contributions, and ensure you have specifically and accurately indicated the role(s) that these authors had in your study. These amendments should be made in the online form. Response: This has been done

b. Confirm in your cover letter that you agree with the following statement, and we will change the online submission form on your behalf: 
“The funder provided support in the form of salaries for authors [insert relevant initials], but did not have any additional role in the study design, data collection and analysis, decision to publish, or preparation of the manuscript. The specific roles of these authors are articulated in the ‘author contributions’ section. -

Response: This language has been included in the cover letter and we have clarified that the seed grant was used for research implementation, data collection and payment of salaries for the research assistants at the study site in Kampala, Uganda. The seed grant was not used to pay salaries for any of the investigators or co-authors.

4. Thank you for stating the following in the Acknowledgments Section of your manuscript: 
"Funding: This work was supported by the University of Georgia internal seed grants program. 
The authors are grateful to Dr. Koichiro Otani for his initial assistance in adopting the Consumer Assessment of Healthcare Providers and Systems (CAHPS) survey instrument for measuring satisfaction. We acknowledge the clinical contribution of Dr. Sarah Zalwango and the help of the research assistants; Damalie Nakkonde, Esther Nakayenga and Joyce Nalubowa in collecting the data, the staff at the HIV clinic, the data clerk and the participants in Kampala, Uganda.
AUTHORSHIP CONTRIBUTIONS AND CONFIRMATION STATEMENT 
Conception and design: JNS, JM, SM, RK data collection: RK, JS data analyses: MEC, HW, JNS interpretation of the study results: JNS, MEC, HW, RK, JM, SM first draft of the manuscript: JNS, MEC. Review and edit of the manuscript: JNS, MEC, HW, RK, SM, JM. All authors approved the final version of the manuscript."

We note that you have provided funding information that is not currently declared in your Funding Statement. However, funding information should not appear in the Acknowledgments section or other areas of your manuscript. We will only publish funding information present in the Funding Statement section of the online submission form.

Response: Funding information has been removed from acknowledgements.
Please remove any funding-related text from the manuscript and let us know how you would like to update your Funding Statement. Currently, your Funding Statement reads as follows: "JNS and KO received an internal seed grant from the University of Georgia.
The funders had no role in study design, data collection and analysis, decision to publish, or preparation of the manuscript."

Please include your amended statements within your cover letter; we will change the online submission form on your behalf.-

Response: The statement has been added to the cover letter.

5. In your Data Availability statement, you have not specified where the minimal data set underlying the results described in your manuscript can be found. PLOS defines a study's minimal data set as the underlying data used to reach the conclusions drawn in the manuscript and any additional data required to replicate the reported study findings in their entirety. All PLOS journals require that the minimal data set be made fully available. For more information about our data policy, please see <http://journals.plos.org/plosone/s/data-availability>.

Upon re-submitting your revised manuscript, please upload your study’s minimal underlying data set as either Supporting Information files or to a stable, public repository and include the relevant URLs, DOIs, or accession numbers within your revised cover letter. For a list of acceptable repositories, please see <http://journals.plos.org/plosone/s/data-availability#loc-recommended-repositories>. Any potentially identifying patient information must be fully anonymized.

Important: If there are ethical or legal restrictions to sharing your data publicly, please explain these restrictions in detail. Please see our guidelines for more information on what we consider unacceptable restrictions to publicly sharing data: <http://journals.plos.org/plosone/s/data-availability#loc-unacceptable-data-access-restrictions>. Note that it is not acceptable for the authors to be the sole named individuals responsible for ensuring data access.

We will update your Data Availability statement to reflect the information you provide in your cover letter.

Response: **Data Availability:** Data cannot be made publicly available due to ethical restrictions. After closer consultation with the research office at the University of Georgia, the IRB approval for this study restricts the sharing of individual-level data. An anonymized dataset is available upon request from researchers who meet the criteria for access to confidential information. Data requests may be sent to the Human Subjects Office Director at University of Georgia, Kim Fowler (phone contact: 706-542-5318, and email contact: [irb@uga.edu](mailto:irb@uga.edu)). In particular, we welcome researchers willing to create a strong data-sharing partnership and collaboration with the Ugandan researchers who generated the data.

6. Please include captions for your Supporting Information files at the end of your manuscript, and update any in-text citations to match accordingly. Please see our Supporting Information guidelines for more information: <http://journals.plos.org/plosone/s/supporting-information>.

**Response to Specific Reviewers Comments**

**Reviewer #1**:

Thank you for the opportunity to review manuscript PONE-D-21-27071 entitled, S Health Provider Skills and Quality of Reception Services Influence Patient Satisfaction Among People Living with HIV Receiving Antiretroviral Therapy in Urban Uganda: A Factor Analysis

The study reports findings from a cross sectional study conducted among people with HIV to evaluate the internal reliability of the CAHPS scale and factors associated with higher patient satisfaction. The paper is generally well-written and well-organized. The description of procedures for translating the instruments and evaluating its internal validity and reliability are generally well-conceived and carried out. The study’s premise could be argued better. It’s not clear why the new instrument is needed. Likewise, the rationale and procedures for adapting the instrument are less clear and instructions for scoring are not clear, which might discourage future use of the scale. The finding that facility satisfaction was higher in those who reported higher satisfaction with receptionists is interesting.

Response: The CG-CAHPS instrument was use because of its focus on capturing patient experiences. The CAHPS tool is unique in that it covers domains of patient experience: accessibility to care, communication with providers, care coordination and interaction with the non-medical staff i.e. receptionists/clerks.

Authors state the “CAHPS has not been assessed in an African population”. While this may be true, it’s not clear why this justifies the study. In arguing the study’s premise and rationale for the current study, it may be beneficial to discuss the research studies that have evaluated patient satisfaction in SSA and the strengths and limitations of those instruments. For example, researchers in Lusaka recently used a 9-item satisfaction scale adapted from the Adult Primary Care Questionnaire: Mukamba N, Chilyabanyama ON, Beres LK…Schwartz SR. Patients’ satisfaction with HIV care providers in public health facilities in Lusaka: a study of patients who were lost-to-follow-up from HIV care and treatment. AIDS and Behavior. 2020 Apr;24(4):1151-60.

Response: There is no standard to measure patient satisfaction, however a variety of scales exist and have been used in HIV-infected populations in the African setting. For example, Mukamba and colleagues (2020) adapted the Adult Primary Care Questionnaire with 9-items that focus specifically on the patients’ interaction with the doctor. In our study, we chose to use the CAHPS instrument because it captures experiences with a broader range health staff including the clerks and receptionists who might indeed influence their satisfaction with the services. Understanding patients experiences from check-in to checkout of the health facility is essential to providing a complete picture of health care quality. Moreover, capturing the non-medical aspects of a patients experience at a clinic visit would ensure that the health quality interventions encompass the full spectrum of touch points.

**Page 4:** The authors discuss their sampling approach and describe using “a sampling interval of five”. One might assume that every fifth patient was invited to participate, however, more details about the process and personnel (clinic staff or researchers) who made the initial contact to recruit subjects and determine eligibility. Also, how was social desirability bias addressed given that participants were being asked to rate the same facility where they received services?

Response: The number of patients to be interviewed were based on the daily average patient load in the study clinic. Based on the records, approximately 113 patients visited the HIV clinic each day. We aimed to recruit 20 patients per day in order to achieve the estimated sample size within the planned study enrollment period. We then employed systematic random sampling of every 5^th^ patients who showed up at the clinic on each day of week, Monday through Friday. The clinic nurses assisted the study to team to identify eligible patients and referred them for brief information about the study and then invited to participate voluntarily. If they declined they were replaced by the next patient who met the interval of 5 sampling procedure.

Response: Although social desirability bias could have possibly occurred given that participants were being asked to rate the same facility where they received services, we minimized it by hiring a separate research team of nurses who do not routinely work at the same HIV clinic. Additionally, patients were assured that their information was to be kept confidential and only shared in a general report as recommendations for improvement of quality of services.

**Page 5:** The authors describe modifying/adapting the CAHPS instrument and selecting 18 questions. What was the rationale for modifying the instrument? It might be useful to mention the number of items in the original instrument and the rational for selecting 18. Also, rather than list the items individually in a paragraph, it might be preferable to have a table. Was mental and emotional health part of the original survey? How were these constructs measured and why were they important to include? What other questions were added or dropped before administering the modified instrument?

Response: Questions #1-5 were modified to suit the context and questions #6- 24 were adapted for use in specifically measuring satisfaction with the domains of interest. A table of all the items adapted from the CAHPS-CG (Table 1) is now shown in the text and a copy of the version of the modified instrument is provided in supporting information as S3.

**Page 5-6:** The formula for scoring the CAHPS is unclear. It’s also unclear whether the authors are using the original scoring formula or their own.

Response: We have used specific guidance from the CAHPS to normalize the scoring of the CAHPS. See link below:

[The CAHPS Ambulatory Care Improvement Guide: Determining Where to Focus Efforts to Improve Patient Experience (ahrq.gov)](https://www.ahrq.gov/sites/default/files/wysiwyg/cahps/quality-improvement/improvement-guide/5-determining-focus/cahps-ambulatory-care-guide-section-5.pdf) see Figure 5-2. Comparison of Practice Site Normalized Mean Scores to Group Normalized Mean Scores

Normalizing is a way to transform all scores to the same scale, typically 0 to 100. It is done to ease comparison across items and composites that use different response scales. To transform the scores, one would first transform the response values at the respondent level from 0-100 using the following formula: Normalized Score = 100*(Respondent’s selected response value – Minimum response value on scale) / (Maximum response value – Minimum response value) For example, the responses on a four-point scale would be normalized as follows: **Response Option** 1, 2, 3, 4 and **Normalized Response** 0.00,33.33, 66.67, 100.00 respectively.

The significance of the patient satisfaction scores are hard to appreciate without an understanding of the scale range and what different values represent. The authors stated previously that the maximum possible score for the “composite index” was 3.94, which is not easily interpreted. For example, it’s unclear what a “2.08” means for “How often did you see a provider within one hour”.

Response: The patient satisfaction scores have been normalized to a scale of 0 to 100 and an arithmetic mean calculated to make it easier for the readers to understand.

It would be interesting to see the actual survey responses. For example, the percentage who agreed or disagreed with each of the 18 items.

Response: The results of responses from the 18 items have been presented in figure 1 and in a table as supporting information see Table S1.

The word “prediction” is used frequently in describing the multivariate modeling and results. Given the study design and the lack of a conceptual model, might be preferable to talk about “associations” in the data. The authors do a good job of acknowledging this in the discussion, but not as much with the results.

Response: This language has been corrected to show that they are associations

From the output in Table 4, it appears that sex and mental health were associated with health facility rating as were patient satisfaction sub-scales. It’s not clear where the authors see evidence of an association with educational level, although this is mentioned in text.
Response: This has been clarified to show that education at senior level was a significant factor at bivariate analysis but was not significant at multivariable regression analysis suggesting it is an independent factor.
Line 280-281: The authors are cautioned to avoid language implying that factor loadings are indicative of the influence of these items of patient satisfaction. The loadings indicate how well the items cluster.

Response: This language has been modified to read as such “Factor loadings specifically show how well the items cluster together. Our findings highlight the importance of the timeliness with reception services and courteous treatment of patients by the clerical staff in the HIV clinics. ” now in line 301- 303.

**Minor
Abstract**
Consider using a comma after the phrase “emotional health”.- **This has been corrected
Manuscript**
line 65 – the “S” in AIDS should be capitalized. - **This has been corrected
Manuscript**
line 66, 67 – capitalization not needed for “Antiretroviral”, “People”, “Living”. **This has been corrected
Manuscript**
line 71 = comma after “Africa” **This has been corrected
Manuscript**
line 113, add the word “as” after “such”. **This has been corrected**
**Manuscript**
line 181, remove the word “and”. **This has been corrected
Manuscript**
Alpha reported variously as 0.92 and 0.94. Check to make sure reliability estimates are reported consistently- **This has been corrected, 0.94 is the overall Cronbach’s alpha number**
