# Supplementary material for: Patient satisfaction among persons living with HIV/AIDS and receiving antiretroviral therapy in urban Uganda: A factor analysis
Source: PLoS One. 2023 Feb 2;18(2):e0280732. doi: 10.1371/journal.pone.0280732 (PMC9894454; doi:10.1371/journal.pone.0280732)
Supplement: Attachment — Submitted filename: Rebuttal Letter_Reviewers comments R2.docx [file pone.0280732.s005.docx]

**Response to Reviewers Comments**

**Reviewer #2:** Thanks for sending me this interesting survey on satisfaction of HIV patients with outpatient clinics. Here are some comments.

This is a revised manuscript and as seen in track-change file, substantial modifications have been carried out. I believe this survey is important especially in developing countries with volatile healthcare atmosphere.

1. The questionnaire name is inconsistently used throughout paper. CAHPS vs CHAPS—

Response: This error has been corrected to be consistent as CAHPS

The introduction is prepared very well

2.The methods sections is very long and many details could be shortened or merged.

Response: This point is well noted and appreciated. However, the authors have agreed to retain the details in the methods section because some content was just added to expand details as part of the first revision in response to comments from other reviewers.

3. The note that about ¾ of participants were female could jeopardize the sampling method. Authors need to mention the baseline composition of whole clinic patients and see if the participants are similarly ¾ of whole pool are females, too.

Response: We acknowledge the possible overrepresentation of female participants in the study sample. However, we note that the demographic distribution of HIV prevalence is Uganda is consistently skewed towards female. In 2018, the Uganda HIV/AIDS progress report showed that the prevalence of HIV/AIDS in women 15+ is 7.6% while that of men is 4.7% in the same age group. Therefore, based on this distribution the proportion of female patients attending HIV/AIDS clinic can be expected to be higher accordingly.

4. Beside the recall bias, sampling bias / selection bias should be mentioned in limitation section too. For instance, patients being less interested in this clinic or those with worse conditions are less likely to participate.

Response: We have added sampling and selection bias as part of the acknowledged possible study limitations

**Reviewer #3:** I would like to thank the editor for providing the chance to review this submission and the authors for their precious efforts. The authors of this study investigated the important notion of patient satisfaction among patients with HIV residing in urban Uganda as a health services research endeavor. The submission benefits from a previous round of review and revision and I am happy to evaluate the R1 draft. In my initial evaluation, the authors were successful in addressing and answering the previous reviewer’s comments in this revision. However, the manuscript could benefit from further changes. My comments and suggestions in this regard on this version of the manuscript are provided below.
1. Lines 1-2: the provided title could be briefer and clearer in transferring the study notion.

The title has been shortened as suggested by the reviewer to read “Patient satisfaction among persons living with HIV and receiving antiretroviral therapy in urban Uganda: A factor analysis”
2. Lines 31-33: this part of the introduction is more a methods part and could be moved to the next part in the abstract and instead the aim of study is suggested to be added in these lines.

This suggested revision has been made. Thank you!
3. Line 35: since none of the authors of this study are affiliated with any institution in Uganda, it should be clear that authors used a pre-collected data or they conducted themselves the data collection for this study.

The first (Juliet N. Sekandi) and fourth (Robert Kakaire) authors, are both native Ugandans who both physically conducted this project during their post-doc and doctoral training respectively in summer time in Uganda. They led the development and data collection while supervising the research assistants. None of the research assistants fully met the requirements to be co-author hence they are listed in the acknowledgements.
4. Lines 43-47: adding some numbers to the provided results in abstract could be beneficial for readers, especially about the significant associations of mentioned factors with patient satisfaction. The p-values to indicate significant associations have been added to the abstract
5. Lines 50-52: before ant general conclusion, providing a sentence driven by the results of this study is necessary to summarize the findings of this investigation. The conclusion has been revised according to the reviewer’s suggestion, Thank you!
6. Introduction: revising this section in order to make the first paragraph talking about the epidemiology and burden of HIV/AIDS in general and in Uganda, and the second paragraph talking about patient satisfaction analysis could be better and is highly suggested. We appreciate this suggestion and we have made some changes. However, we note that the current version of the introduction was set up in that format based on a previous revision suggested by another reviewer. To avoid any confusion the authors have tried to strike a balance in the revisions.

7. Line 122: it should be made clear all through the text that this study recruited all HIV/AIDS patients and not only those in HIV stage. This is vague in the current format of presentation.

This presentation has been corrected to HIV/AIDS consistently throughout the manuscript.
8. Lines 219-221: the authors may add about the logic of choosing independent variables for the regression analysis. Were the mentioned variables included based on a literature review or by significant results of a primary bivariate regression analysis? The details of these steps should be added to this part of the methods. Yes, we based primarily on the significant variables at bivariate analysis and as the literature review including our study in Kampala as the second guide to selection. This has been added to the manuscript
9. Line 238: adding the details of statistical measures like mean and SD used to report the results to the methods could be useful. These are already included
10. Discussion: this section needs a paragraph on the practical implications of this study findings and some points for local and regional health authorities to benefit the most by the conducted efforts. A short paragraph on practical implications has been added to the discussion section
